# Clozapine for Treatment Resistant Aggression in Autism

**DOI:** 10.1192/bjo.2022.374

**Published:** 2022-06-20

**Authors:** Abi Williamson, Vanathy Raja, Lesley Bailey

**Affiliations:** Mersey Care NHS Foundation Trust, Liverpool, United Kingdom

## Abstract

**Aims:**

The National Institute for Health and Care Excellence (NICE) guidance on the management of behaviour that challenges in autism, is that medication should be considered when psychosocial or other interventions cannot be delivered because of the severity of the behaviour. In our experience of working in Secure and Specialist Learning Disability, there are also times when challenging behaviour continues despite non-pharmacological interventions being optimised. There is (limited) evidence that clozapine should be considered for the management of aggression in patients with autism not improved by first-line antipsychotic drugs.

**Methods:**

We present two cases of female patients with autism and learning disability, both of whom had been detained for a long period under the Mental Health Act 1983. Both continued to present with significant aggression despite non-pharmacological treatment being optimised. The aggression did not respond to first-line antipsychotic drugs, nor other psychotropic medication. They were started on clozapine.

In the first case, that of a 32-year-old, aggressive incidents reduced from a mean of 15 per month to 5 per month. The use of physical restraint reduced from 10 episodes per month to 5 per month. Staff reported that aggression was less severe than previously. Due to the improvement, the patient began having access to escorted community leave.

In the second case, that of a 31-year-old, incidents of aggression requiring floor restraint reduced from a mean of 30 episodes per month to 15 per month. The average monthly duration of restraint reduced from 29.5 minutes to 18.5 minutes. Although difficult to quantify, the staff team consistently reported that her level of arousal at times of incidents was less. Her engagement levels also increased. She became more tolerant of people being in her living space and actively sought out contact with staff.

**Results:**

Clozapine resulted in a reduction in aggression and arguably, improved quality of life, for the two patients described. We make recommendations on when clozapine could be considered for treatment resistant aggression in autism and what should be done before this. We also provide guidance on how a therapeutic trial should be conducted, in line with Stopping Over Medication of People with a Learning Disability, autism or both with psychotropic medicines (STOMP-LD).

**Conclusion:**

It is reasonable to consider clozapine for aggression in autism when all other interventions have failed. It may result in meaningful change and improved quality of life.

